# Paget's Disease of the Vulva in Premenopausal Woman Treated with Only Surgery: A Case Report

**DOI:** 10.1155/2012/854827

**Published:** 2012-09-16

**Authors:** Hamid Asmouki, Rachid Oumouloud, Abderrahim Aboulfalah, Abderraouf Soummani, Abdelouahed Marrat

**Affiliations:** ^1^Department of Obstetrics and Gynaecology, School of Medicine and Pharmacy, Mohammed VI University Hospital, Cadi Ayyad University, Marrakech 40000, Morocco; ^2^Al Fadle Histopathology Laboratory, Amitaf Residence, Avenue Yaacoub El Mansour, Appartment no. 5, Marrakech 40000, Morocco

## Abstract

Paget's disease of the vulva remains a rare condition with only a limited number of cases reported in the literature. It is an uncommon neoplasm usually of postmenopausal white women characterized by controversies in its prevalence, clinical features, treatment strategies, and prognostic. We here report a case of a primary Paget's disease of the vulva in premenopausal woman treated by only surgery with a favorable issue.

## 1. Introduction

 In 1874, Sir James Paget published the first description of the disease, his original case was of disease involving the nipple and areola [1]. Fifteen years later (1889), Crocker reported extramammary Paget's disease affecting the scrotum and penis [2]. In 1901, Dubreuilh described the characteristic “cake-icing appearance” of vulvar Paget's disease [3]. This very rare malignancy is originating in vulvar apocrine-gland bearing skin cells or as a manifestation of adjacent primary anal, rectal, or bladder adenocarcinoma [4]. It is an uncommon disease with incidence reports varying between <1% and 2% of vulvar malignancies [5]. Paget's disease of the vulva (PDV) is commonly seen in postmenopausal Caucasian females; and appears clinically as red, eczematous, and pruriginous—and sometimes painful—lesions [6]. Surgery has been the treatment of choice, but because of the disappointing results of surgical treatment, many authors have been encouraged to try other procedures for PDV. 

 We will discuss the diagnostic features, treatment, and prognosis of this disease through the observation of a 43-year-old woman with noninvasive PDV treated by only surgery.

## 2. Case Presentation

 A 43-year-old nonmenopausal woman presented to department of dermatology with complaints of vulvar pruritus, painful vulvar lesion, and recurrent vaginal discharges, during one year.

 She was treated with topical steroids and antimycotics. As there was no improvement with the above treatment, she was referred to department of obstetrics and gynecology.

**Figure 1:**
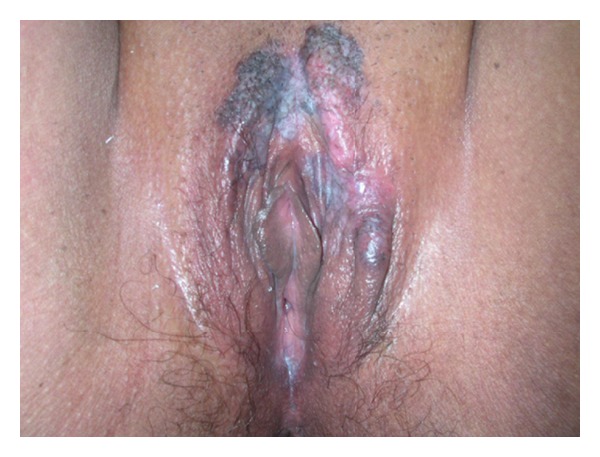
Macroscopic appearance of Paget's disease of the vulva—erythemato-squamous lesion extended to the labia majora and labia minora.

 On examination, patient had papular and erythemato-squamous vulvar lesion extended to the labia majora and labia minora (Figure 1). Cervical exam was normal. Vulvar biopsy was taken, histopathological examination showed noninvasive PDV. The treatment was surgery with wide local resection and margin control by frozen section examination. No recurrence was seen during two years after treatment with disappearance of pain and improved quality of life.

## 3. Discussion

 Paget's disease of the vulva (PDV) is a rare locally recurrent chronic disease, accounting for less than 1% of vulvar neoplasms [7]. The vulva remains the most frequently involved site with 65% of extramammary Paget's disease located in this area [8]. It occurs predominantly in postmenopausal Caucasian women [6]. The rarity of this disease has caused difficulties in its characterization. Controversies exist in the literature regarding its pathogenesis, the prevalence of concurrent underlying adenocarcinoma, associated malignancies, optimal treatment, and recurrence after surgical excision. 

 Concerning the pathogenesis, it is still the subject of great debate. Toker cells have been described as precursor cells of both mammary and extramammary Paget's disease. These cells are found in the basal layer of the epidermis and are adjacent to the lactiferous ducts in the nipple [9]. They also occur as a normal constituent of genital skin in association with mammary like glands of the vulva [10]. Another theory has been advanced, the concept that Paget cells are in fact malignant keratinocytes, which has been transformed in situ [11]. Current evidence supports the fact that angiogenesis plays an important role in the pathogenesis of PDV. However Ellis et al. [12] demonstrated that it is possible that in PDV, Paget cells can migrate and progress to invasive disease by utilizing the existing vasculature, without the need for the formation of new blood vessels. 

 Clinically, the lesions in PDV are non specific and multiple topical therapies are often tried before the diagnosis is made, a median delay of two years has been reported [5, 13]. It appears as a pink eczematoid area with white islands of hyperkeratosis that is accompanied by pruritus in 70% of patients [14]. A palpable mass should raise concern for underlying invasive disease. A classification system has been proposed by Wilkinson and Brown that divides vulvar Paget's disease into two groups, primary and secondary disease. Primary cutaneous Paget's disease is an intraepithelial adenocarcinoma arising within the epidermis or underlying skin appendages. Secondary or noncutaneous Paget's disease originates from an underlying noncutaneous adenocarcinoma, most commonly anorectal adenocarcinoma, and urothelial carcinoma of the bladder or urethra, carcinoma of the cervix, ovary, or endometrium [15]. The diagnostic is established by a biopsy, and immunohistochemical studies may be helpful in distinguishing primary and secondary lesions [16].

 The usual treatment for PDV is surgical excision. Over the years, many therapeutic modalities have been attempted on patients with PDV in an effort to reduce the significant morbidity associated with the often-radical surgical treatments performed. In case of invasive PDV, wide partial or total vulvectomy with inguinal-femoral nodal assessment followed by chemotherapy or radiotherapy is advised. Because of the high recurrence rates ranging between 30–60% and attributed to subclinical extension and multifocal disease, margin status gained interest [17] with intraoperative frozen section analysis [18]. In our case, there was negative surgical margin which can explain absence of recurrence. Major controversy exists regarding the influence of surgical margins status on the recurrence rate. Some studies found that positive margins correlate with increased recurrence rate [19, 20]. However, other studies have found no correlation between margin status and local recurrence [21–24]. The high frequency of recurrence, usually outside the area of previous resection, remains the most challenging feature in the management of vulvar intraepithelial Paget's disease. Long-term monitoring of patients is recommended, and repeat surgical excision is often necessary [24]. 

 Alternative nonsurgical or combined treatments have been proposed, including radiotherapy, topical chemotherapy with intralesional interferon alfa-2b, laser ablation, photodynamic therapy, topical fluorouracil (5-Fu), and imiquimod 5% cream [25]. Radiotherapy has been used both as a definitive treatment as well as in the postoperative setting to prevent local recurrence. It should be used as an initial treatment mostly in selected patient groups (elderly, those unfit to undergo conventional surgery), as well as postsurgical treatment in positive surgical margins or recurrent disease [26]. While some state that radiation therapy produces poor results [27], others disagree [19, 28]. Laser treatment of PDV gained interest in the hopes that it would provide a conservative approach to eradicate the disease while preserving vulvar anatomy and sexual function. This procedure has been used successfully [29, 30], but has the disadvantage of significant postoperative pain and high recurrence rate [19, 31]. Another option for clinical treatment of PDV, topical imiquimod 5% cream seems to be promising [25]. Topical photodynamic therapy (PDT) offers an optional approach for extramammary Paget's disease. The complete clinical response rate to PDT at month 6, after two series of two illuminations, was equivalent to that for surgery. Although the recurrence rate was high, this treatment may be repeated without functional or physical consequences. PDT resulted in disappearance of pain and improved quality of life [32]. In invasive or noninvasive PDV, HER-2/neu protein is found to be overexpressed in 5% to 80% of cases. The availability and success of targeted therapies in the treatment of HER-2/neu positive breast cancer had led some authors to suggest its use in vulvar Paget's disease. Significant improvement of symptoms and significant reduction of vulvar Paget's lesion were reported in recurrent disease treated with Trastuzumab [33]. No adjuvant treatment was used in our case because of the favorable issue of the disease.

## 4. Conclusion

 Despite Paget's disease of the vulva has been described over 100 years ago, still remains a largely confusing and relatively rare disease. It is clear that randomized controlled trials are needed to adequately evaluate and compare therapeutic modalities, so that standardization may be attempted and initial treatment protocols elaborated to determine the best combination of greatest efficacy with least morbidity.
